# Collective Empowerment in Online Health Communities: Scale Development and Empirical Validation

**DOI:** 10.2196/14392

**Published:** 2019-11-20

**Authors:** Sara Atanasova, Gregor Petric

**Affiliations:** 1 Centre for Methodology and Informatics Faculty of Social Sciences University of Ljubljana Ljubljana Slovenia

**Keywords:** patient empowerment, collective empowerment, online health community, psychometrics, reliability, validity, weights and measures

## Abstract

**Background:**

The role of online health communities (OHCs) in patient empowerment is growing and has been increasingly studied in recent years. Research has focused primarily on individualistic conception of patients’ empowerment, with much less attention paid to the role of OHCs in the development of patients’ collective empowerment. Although OHCs have immense potential for empowerment that goes beyond the individual, the concept and scale of collective empowerment in OHCs have not yet been developed or validated.

**Objective:**

This study aimed to develop an instrument for measuring collective empowerment in online health communities (CE-OHC) and to test its quality by investigating its factorial structure, reliability, construct validity, and predictive validity.

**Methods:**

The CE-OHC scale was developed according to a strict methodology for developing valid and reliable scales. An initial set of 20 items was first tested in the pilot study conducted in 2016 using a sample of 280 registered users of Slovenia’s largest OHC. A refined version with 11 items was tested in the main study conducted in 2018 on a random sample of 30,000 registered users of the same OHC. The final sample comprised 784 users. Exploratory factor analysis (EFA) and confirmatory factor analysis (CFA) were used to investigate the factorial structure, discriminant validity, and convergent validity of the scale. Cronbach alpha coefficient was used to determine the CE-OHC scale’s internal consistency. To establish the predictive validity, ordinary least squares regression was performed to test the role of CE-OHC in users’ civic participation.

**Results:**

The EFA resulted in a two-factor solution, and the two factors—knowledge of resources and resource mobilization for collective action—together explain 63.8% of the variance. The second-order CFA demonstrated a good fit to the data (root mean square error of approximation=0.07) and the scale had a good internal consistency (alpha=.86). Although evidence of the scale’s convergent validity was partially provided, discriminant validity of the scale remained unconfirmed. Overall, CE-OHC was confirmed to be a predictor of users’ civic participation, but the influence was somewhat weak and inconsistent across two subscales.

**Conclusions:**

The proposed CE-OHC scale is a reliable and relatively valid instrument and serves as a good baseline to advance the measurement of collective empowerment in OHC contexts. This is the first scale developed for this purpose, and future research should focus on the development of a clear nomological network of the collective empowerment construct in relation to the OHC settings.

## Introduction

### Online Health Communities as Platforms for Patient Empowerment

Online health communities (OHCs) are among the most important electronic health (eHealth) services in contemporary society [1]. OHC users, who are usually patients, caregivers, or other individuals interested in health-related issues, can search for and exchange health-related information, experiences, advice and social support, and/or influence public opinion and interact with other users and health professional moderators (usually doctors and health care providers), or simply observe others’ interactions [1-9]. Several studies have demonstrated that the various activities of OHC users lead to patient empowerment [5,10,11], which is manifested in various positive outcomes for OHC users: higher self-esteem, self-efficacy, and control related to the management of one’s health issues; enhanced satisfaction from helping others; improved confidence in interaction with doctors; more competent use of health services; and even enhanced social well-being and quality of life [6,12-15]. It is thus unsurprising that empowerment has become one of the central concepts within OHC studies. The importance of OHCs in patient empowerment has been increasingly acknowledged and studied in recent years; however, research has been primarily concerned with patients’ individual empowerment, with little consideration of the role of OHCs in the development of patients’ collective empowerment.

### Lack of Research on Collective Empowerment

Patient empowerment is a predominately individualistic concept, originating from the general cultural shift in Western cultures toward individualism and consumerism in health care and focuses on various domains pertaining to patient, such as patient states and experiences, action and behaviors, self-determination, and skills [16]. Consequently, the research field is rich in measurement instruments that tap on some or all of the mentioned domains (refer to the study by Barr et al [16] for systematic review of such scales). Studies in health and health care, and OHCs specifically, focus almost exclusively on individual empowerment [13,17]. This is unsurprising as it has been demonstrated that if individuals experiencing health problems have positive attitudes, confidence, and other abilities required to manage their health, they may expect better health outcomes than individuals who are disengaged, apathetic, and resigned [18]. However, research of OHC suggests that at least in the context of this type of Web-based platforms, patients can also experience empowerment that is of intersubjective nature—called collective empowerment [6,15]. This finding is not specific to OHC research as the collective empowerment was already emphasized as important component of empowerment by the general empowerment theory from the field of community psychology. This theory clearly suggests that empowerment consists of at least 2 dimensions [19-22], that is, *individual* or *intrapersonal empowerment* and *collective empowerment* (also often referred to as *interactional* or *cognitive empowerment*) [23,24]. The domains of individual empowerment—such as abilities to develop a sense of control over personal health, self-efficacy, and competence in managing health conditions [20,25]—are clearly reflected in the concept of patient empowerment. Conversely, the ideas of developing psychological capacity for initiation or support of (potential) changes in social circumstances that affect patients’ health conditions and the accessibility and quality of health services or health care system in general [20,26] are much less present in discussions of patient empowerment. This is considered by the concept of collective empowerment, which pertains to the individuals’ beliefs that personal health-related issues can be (effectively) solved in collaboration with others and by enacting influence in wider social structures collectively [20,27].

The concept of collective empowerment is very relevant, at least in the context of OHCs, as these platforms allow users to engage in discussions of health politics and topics related to their lifestyles and values as individuals or groups. Moreover, social identity theory suggests that social identity processes in OHCs drive participatory behaviors and users’ identification with the community, leading to users’ collective engagement [28]. OHCs can thus function as communicative spaces in which, as in other types of online communities, participants may collectively engage and increase their social power as an interest group, with the aim of influencing the institutionalized arrangements and political decisions that affect their quality of life [29-31].

OHCs have indeed become an important arena in which individuals, patients, caregivers, and groups may voice their stances that challenge health policy, belief systems, practices in health care institutions, and services [32]. The bottom-up collective engagement that OHCs facilitate addresses topics that include access to or provision of health care services, health inequality, disease prevention and illness advocacy, health care reform, patients’ rights, and power relationships in the health care arena. Moreover, OHCs often function as platforms for discussion and exchange of information related to the accessibility of remedies and medical treatments; access to health care services and health care professionals; misconceptions of specific, often stigmatized illnesses such as AIDS/HIV, infertility, and mental disorders; and other disease-related issues that often pertain to the disadvantaged social positions of specific patient groups [33,34].

### Aim of the Study

The concept of collective empowerment has been studied in community psychology research [35,36], social identity theoretical perspectives [37,38] and, to some extent, implicitly investigated through concepts of patient engagement and activation in health studies [25,39]. However, studies of OHCs have investigated collective empowerment to a very limited extent [6,15,27,30,40-42] and, empirically, they offer no measurement instruments for assessing collective empowerment in OHCs. Hitherto, collective empowerment scales have been developed in community psychology research [36,43,44] and online community studies [6,30], but they have not been properly adapted to health-related contexts on the Web and empirically validated for the OHC setting. To develop a valid measure of collective empowerment in OHCs, the scale must consider the specific context of OHCs, which, compared with general online communities and other contexts, cover topics including personal health issues and discussions of health care services and the health care system. As Zimmerman observed [20], the concept of empowerment is contextually dependent, varying across different populations and settings. Thus, it is crucial that any instrument used to measure collective empowerment is appropriately adapted to the relevant setting. Therefore, the aims of this study were (1) to develop a valid instrument for measuring collective empowerment in online health communities (CE-OHC) and (2) to test this instrument’s quality by investigating its factorial structure, reliability, construct validity, and predictive validity.

### Defining Collective Empowerment in Online Health Communities and Establishing Its Construct and Predictive Validity

#### Two Dimensions of Collective Empowerment

Collective empowerment has been generally defined as individuals’ critical awareness and understanding of the sociopolitical environment [19,20] and thus consists of 2 main dimensions: (1) *knowledge of resources* and methods that can be used to impact social change and (2) *resource mobilization for collective action*.

*Knowledge of resources* refers to the application of individuals’ knowledge and competences that might be used to collectively initiate change [15]. As already emphasized by early empowerment theorists [19,20], knowledge of resources comprises a critical assessment of individuals’ social and political source(s) of their problem and the development of strategies aimed at collectively overcoming obstacles to achieving their goals. Web-based health-related settings such as OHCs play an important role in patients’ processes of acquiring knowledge of the actions, strategies, or assets needed and applying this knowledge to address health-related problems. This knowledge may be acquired through user interactions whereby (collective) resources may be identified, potentially leading to collaborative efforts to develop strategies and solutions aimed at overcoming limitations in the issues affecting their health. For example, a qualitative study by Ammari and Schoenebeck [40] that explored online support groups for parents of children with special needs demonstrated that these parents were likely to connect, interact, and share their knowledge with other parents and to provide one another with insights into practices and strategies for addressing their child’s health-related problems. Often, such collective efforts pushed parents to embrace advocacy beyond their own children’s needs, leading to the development of interest and active individual and collective participation in legal, policy, and budgetary issues pertaining to their children’s health conditions [40]. Without the knowledge of the resources required to resolve a specific problem that affects not only one individual but pertains more broadly to higher-order social structures, it is highly unlikely that individuals will be motivated to mobilize and influence the challenging social circumstances in a collective effort [45].

The second dimension of collective empowerment, *resource mobilization for collective action*, relates to individuals’ awareness of the possibility for collective engagement and, with other individuals, the collective influence of arrangements in the specific social setting [19,45]. This dimension addresses individuals’ recognition of the need for collaboration and coordination among larger groups—for example, community members—and for strengthening interpersonal relationships to exert an impact on the wider social circumstances that affect their lives and place them in a disadvantaged position [19]. OHCs have been shown to offer an important platform for the development of collective awareness and engagement that unite their members in a belief that personal health-related issues can be effectively solved through collaboration with others and by enacting collective influence in wider social structures [27,46].

#### Construct Validity

Construct validity is one of the most important indicators of a measurement instrument’s quality as it pertains to the extent to which items in the scale actually measure what they are supposed to measure [47]. One common way to empirically assess construct validity is to investigate whether the proposed measure *behaves* as it should in relation to established measures of other constructs from the field. Empirically, construct validity is thus established via *convergent* and *discriminant validity*. The former refers to an empirical similarity between measures of theoretically related constructs and the latter pertains to the absence of correlation between measures of constructs that are theoretically unrelated [47].

On the basis of the theoretical and empirical evidence reported in previous studies, we identified 3 concepts that scholars emphasize to be correlated with collective empowerment (in OHCs) [19,27,30,41,48]: *sense of (virtual) community*, *involvement in community organization,* and *intensity of participation (in OHCs)*.

The crucial role that the sense of community plays in the development of collective empowerment was emphasized in early studies of empowerment in the field of community psychology [19,44,45]. In OHCs, a *sense of virtual community* is based on the users’ identification with the online community, the perception of influence and emotional connection, and users’ integration into the online community [49]. A sense of virtual community presents a key mechanism in building interpersonal relationships and developing awareness among online community members as it helps them to realize that their collaboration is essential for increasing social power as a group that can influence wider social structures [30]. This has also been emphasized by the social identity theory, which argues that identification with community as an important dimension of a sense of virtual community importantly leads to (effective) coordination, collaboration, collective action of members, and thereby, their collective empowerment [37]. The association between a sense of virtual community and collective empowerment in OHCs has been demonstrated by the studies by 
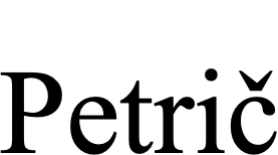
 and 
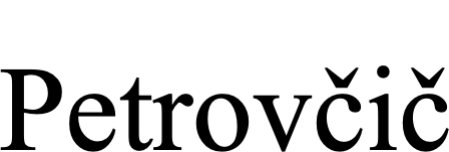
 [27,30]. These studies demonstrated that a sense of virtual community plays a crucial role in building collective empowerment in online communities as it helps users to develop responsibility for the community and a willingness to participate in supportive efforts. It also engenders a sense of social cohesion that encourages community members to collectively organize, develop a common goal, and engage in efforts to achieve it.

In addition, studies from the field of community psychology [19,45], as well as studies on online communities [30,48], attest to the importance of participation in a community’s activities for the development of collective empowerment. *Involvement in community organization* pertains to online community members’ inclusion in discussions about events, vision, and strategies of the online community [50,51]. Active engagement in community organization provides individuals with opportunities to learn new skills, interact with other members, identify needed resources, and develop critical awareness of one’s environment [52].

There is also evidence that collective empowerment is associated with *different forms and intensities of participation in OHCs*. As the empowerment theory suggests, a certain investment in participation and active behavior is required to become empowered [20]. Thus, it is expected that users who contribute more to OHCs, post messages, and interact with other users (ie, posters) experience greater benefits and positive outcomes than users who participate passively in OHCs (ie, lurkers) or do not participate at all. The findings by 
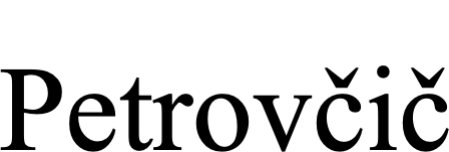
 and 
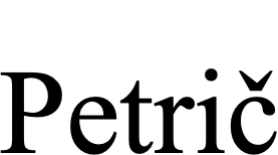
 [6] and Li [41] indicate that participation plays an important role in collective empowerment as it has been confirmed that posters experience a higher level of collective empowerment than lurkers in OHCs.

To investigate discriminant validity, we examined the relationship between collective empowerment in OHCs and *received offline emotional support.* As collective empowerment in OHCs emerges with active participation in the Web-based platforms and the establishment of interpersonal relationships between users, there should be no association between the development of OHC users’ collective empowerment and received offline emotional support. Users’ development of collective empowerment in OHCs highly depends on internal (online) cohesiveness, group identification, and common goals that can lead users to seek political power together to advocate for social change [53], a process in which received offline emotional support should play no part. Moreover, received offline emotional social support leads to the fulfillment of sympathetic and caring behaviors, which is theoretically unrelated to the development of individuals’ critical awareness, understanding of the sociopolitical environment, and knowledge of resources needed to initiate social change and collective action [54].

On the basis of the above theoretical and empirical underpinnings, we proposed the following hypothesis:

H1: Collective empowerment in OHCs is associated with a sense of virtual community, involvement in community organization, and intensity of participation in OHCs, but it is not associated with received offline emotional support.

#### Predictive Validity

To establish predictive validity, the instrument used to measure a latent construct must be evaluated in terms of its ability to predict a certain form of behavior [55]. In our case, predictive validity pertained to the ability of the CE-OHC instrument to predict specific outcomes, such as individuals’ actual participation in a wider sociopolitical environment and, thus, involvement in activities including petitions, demonstrations, and advocacy related to the issues of health-related public concern. In accordance with the empowerment theory [19,20], collective empowerment in OHCs should result in health-related *civic participation*.

Collective empowerment in OHCs refers to the development of a shared understanding among users of their position (as patients) in the wider social domain and leads to collective efforts in challenging existing health care institutions and services. An indirect link, at least, has been observed between collective empowerment and health-related civic participation in the case of an online support group for breast cancer patients in New Zealand. These patients, through participation in the online support group, identified an important issue regarding a national health insurance plan that did not cover a new treatment that, although expensive, was more efficient. With collective engagement and action in an online support group, these patients brought about a change in the national health insurance plan that introduced cover for new breast cancer treatments [33]. To establish the predictive validity of collective empowerment, other important factors of civic participation that have already been empirically validated must also be taken into account; these include a sense of (virtual) community [56], involvement in community organization [57], and intensity of participation in community settings [58]. On the basis of the above, we proposed the second hypothesis:

H2: Collective empowerment in OHCs is a significant predictor of civic participation.

## Methods

### Sample

A pilot study and a main study were conducted to test the proposed CE-OHC scale. Both studies were based on data collected through a self-selected Web-based survey using probability samples of registered users of Slovenia’s largest OHC, Med.Over.Net (MON). MON was founded in 2000 and covers areas of health, medicine, social work, law, and education. It is one of Slovenia’s most visited online communities (and websites), with over 400,000 monthly visits and, on average, over 70,000 monthly users. In May 2018, this OHC had around 150 online discussion forums, around a million and a half forum threads, almost 12 million published forum posts, and around 120,000 registered users. The surveys for both studies were conducted using an open Web survey app 1KA (Eng. *One Click Survey*) developed by Centre for Social Informatics at Faculty of Social Sciences, University of Ljubljana. 1KA has mechanisms that disallow multiple entries by the same respondent. The data collection procedure and samples used in both studies are detailed in the following subsections.

#### Pilot Study

In preparation for the main study, a pilot study was conducted in June 2016 as part of the annual cross-sectional Web-based survey study on MON and its users. The Web survey was administered by the OHC provider and followed all ethical standards for the administration of scientific surveys. The Web survey was conducted on a random sample of 15,000 registered users, and 13.04% (280/2147) of the respondents provided answers to the CE-OHC scale items. Details on the pilot study’s data collection and sample may be found in the study by 
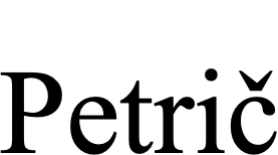
 et al [59].

#### Main Study

The Web-based survey data collection for the main study was incorporated into an annual survey on users’ experiences and satisfaction with the OHC MON, administered between April 25 and May 10, 2018, by the OHC provider and in line with the ethical standards for the administration of scientific surveys. The OHC provider designed a random sample of 30,000 registered users from the list of all registered users. Potential respondents were invited to participate in the Web survey via the OHC’s email newsletter service. The invitation included a description of the study’s purpose and brief information about respondents’ rights and the survey length.

Out of approximately 30,000 potential respondents, 2314 (7.71%) clicked on the link for the Web survey, and 1762 respondents viewed the introduction page with informed consent and clicked the *Next* button to begin the survey. Of these, 676 (38.37%) partially completed and 893 (50.67%) fully completed the survey questionnaire, which led to a 76.15% (1762/2314) completion rate. The total response rate of 5.87% (1726/30,000) is small but not unusual in probability list–based Web surveys, which are long and include sensitive topics [60]. The survey questionnaire took on average 21 min and 33 seconds to complete. After the data screening and cleaning procedures, the final sample comprised 1123 respondents. The analyses were performed on a subsample of 784 respondents who had provided answers to the CE-OHC scale. Missing data were handled with the multiple imputation procedure. More information about the Web survey can be found in the Checklist for Reporting Results of Internet e-Surveys in Multimedia Appendix 1.

The sample consisted of 17.7% (139/784) males and 82.3% (645/784) females (Table 1). The respondents’ average age was 41.1 years (SD 11.5). More than half of the participants of the study had completed higher education (64.7%, 507/784), were employed (72.2%, 566/784), and were married or de facto married (79.5%, 623/784). In the survey, the respondents were asked to self-asses how they perceived their current health on a scale of 1, poor, to 5, excellent. Most respondents reported having good (44.1%, 346/784) or very good (32.1%, 251/784) health status (Table 1).

**Table 1:** Sample characteristics (n=784).

Variable		Value, n (%)
**Gender**		
	Male	139 (17.7)
	Female	645 (82.3)
**Education**		
	Lower	59 (7.5)
	Middle	218 (27.8)
	Higher	507 (64.7)
**Employment status**		
	Employed or self-employed	566 (72.2)
	Unemployed	75 (9.5)
	School-aged youth or student	49 (6.3)
	Retired	49 (6.3)
	Homemaker or caregiver	34 (4.3)
	Other	11 (1.4)
**Marital status**		
	Married or de facto married	623 (79.5)
	Single, divorced, or widowed	161 (20.5)
**Health status**		
	Poor	8 (1.0)
	Fair	72 (9.2)
	Good	346 (44.1)
	Very good	251 (32.1)
	Excellent	107 (13.6)

### Ethical Consideration

The authors of this study had no access to respondents’ emails and received an anonymized dataset that included no identifiable personal information. No institutional ethics approval was required as this was a retrospective study. At all stages of the research process, we carefully protected all collected (personal) data and ensured participants’ anonymity and confidentiality. The pilot and the main studies were also conducted in line with the Code of Ethics for Researchers at the University of Ljubljana [61] and the World Medical Association Declaration of Helsinki on ethical principles for medical research involving human subjects [62].

### Measures

#### Collective Empowerment in Online Health Communities Scale

The scale’s development followed the established process of operationalization from theoretical definition to development/adoption of items and empirical evaluation of the resulting scale [47,63]. By following a strict methodology for developing valid and reliable scales [47], and based on the identified dimensions of collective empowerment, we developed an initial set of 20 items. This item set was evaluated for content validity by 3 experts (1 in social science methodology, 1 in health communication, and 1 in internet studies), and a refined set of 15 items was selected, including 8 items measuring knowledge of resources and 7 items measuring resource mobilization for collective action. For knowledge of resources, 3 items were adopted from the study by Akey et al [64] and 5 were newly developed by the authors. The item set for *resource mobilization for collective action* comprised 2 items adopted from the study by Akey et al [64] and 7 items adopted from the Cognitive Empowerment Scale [19,45]. All items included in the final set for the CE-OHC scale were adapted and modified for the health-related and OHC contexts. The initial item set was pretested and evaluated in the pilot study. On the basis of the results of the pilot study (see Testing the Measurement Model: Pilot Study), we omitted 4 items, and the final version of CE-OHC scale included 11 items—6 (CE-OHC1 to CE-OHC6) for measuring *knowledge of resources* and 5 (CE-OHC7 to CE-OHC11) for measuring *resource mobilization for collective action*. All items were measured using a 5-point Likert-type scale, ranging from 1, strongly disagree, to 5, strongly agree.

#### Sense of Virtual Community

Sense of virtual community was measured by adapting 7 items from the Sense of Community Index [65,66] to the online community context. Respondents were asked to evaluate statements about the OHC forum that they most often visit on a scale of 1, strongly agree and 0, strongly disagree. The scale demonstrated acceptable internal consistency (alpha=.75).

#### Intensity of Participation in the Online Health Community

Intensity of participation in the OHC was measured with 9 items, which asked respondents to assess the frequency of their participation in online forum discussions (eg, posting, commenting, asking questions, opening new forum threads, and encouraging discussion) in the last 12 months on a 5-point scale of 1, never, to 5, very often. Responses to these items were summed in an index demonstrating good internal consistency (alpha=.91).

#### Involvement in Community Organization

Involvement in community organization was measured with 6 items. Respondents were asked about their engagement in activities relating to the OHC’s vision, goals, and internal events. Answers were indicated on a 5-point scale of 1, never, to 5, very often. The 6 items were summed in an index that demonstrated good internal consistency (alpha=.95).

#### Civic Participation

Civic participation was measured with 4 items, asking respondents about their participation in activities related to initiatives and actions in the OHC that pertain to issues of public concern. Respondents indicated their answers on a 5-point scale of 1, never, to 5, very often. Civic participation items were summed in an index that demonstrated good internal consistency (alpha=.83).

#### Received Offline Emotional Support

To measure *received offline emotional support*, 3 items were used to ask respondents, on a 5-point scale of 1, never to 5, very often, how regularly they received various forms of emotional support from people in their everyday lives. This variable was computed as an aggregated average of its items and demonstrated good internal consistency (alpha=.92).

#### Control Variables

*Membership length* was measured by asking respondents to indicate how long they had been users of the OHC on the scale of 1, less than 1 month; 2, less than a year; 3, 1 to 3 years; and 4, more than 3 years.

### Statistical Analyses

To test the measurement model of the CE-OHC scale and to ensure its construct validity [67], we first computed the Kaiser-Meyer-Olkin (KMO) measure of sampling adequacy and Bartlett test of sphericity (BTS) to determine whether our data were suitable for exploratory factor analysis (EFA). The KMO index ranges between 0 and 1, and values above 0.5 are considered suitable for factor analysis [67]. To ensure the suitability of factor analysis, BTS should be statistically significant (*P*≤.05), which indicates that sufficient correlations exist among the variables [68]. EFA was conducted to determine which of the scale’s items should be retained. Factors were extracted using principal axis factoring, which uses estimates of communalities on the diagonal in the extraction process [68]. As we did not expect an orthogonal factor solution, oblimin rotation was used.

Confirmatory factor analysis (CFA) was used to determine how well the measurement model fit the observed data [69]. As the construct of collective empowerment is composed of 2 latent dimensions, we used the second-order CFA approach to establish the construct validity of the CE-OHC scale. Second-order CFA is equivalent to *ordinary* first-order CFA, with the difference that each latent dimension is modeled as an indicator of the second-order single latent construct [70]. In the case of 2 first-order factors, the model is underidentified [69]. To avoid the identification problem, the model requires additional information, which may be accomplished by including a constraint that sets the 2 factor loadings of the latent dimensions equal to one another [69]. To assess the model fit, the following absolute and incremental fit indices were used: (1) root mean square error of approximation (RMSEA, 0.08 as a cutoff for poor fitting models); (2) standardized root mean square residual (SRMR), where a value of less than 0.08 is generally considered a good fit; (3) comparative fit index (CFI), which ranges between 0.0 and 1.0, where values closer to 1.0 indicate good fit (CFI≥0.90); and (4) Tucker Lewis Index (TLI), which also ranges between 0.0 and 1.0, and where TLI≥0.9 indicates a good fit [71].

Construct validity was also assessed using a hypothesis-testing approach by which conceptual framework (theory) underlying the measure is used to state hypotheses regarding the relation between the measure and theoretically (un)related concepts and based on the empirical analysis and findings make inferences whether the measure is valid [72]. Correlation analysis with the Pearson correlation coefficient was used to test the association among CE-OHC, its subscales, and (un)related theoretical measures. The scale’s reliability was assessed with the Cronbach alpha coefficient, which ranges between 0 and 1.0. The rough guidelines are that a value of .7 or higher indicated acceptable reliability and a value of .8 or better indicates good internal consistency [47].

To establish the scale’s predictive validity, ordinary least squares (OLS) regression was performed to test the role of collective empowerment in OHCs in users’ civic participation. Data were analyzed using IBM SPSS and R software, with the *lavaan* package [73] used for second-order CFA.

## Results

### Testing the Measurement Model: Pilot Study

Descriptive analysis of the CE-OHC items demonstrated that the majority are approximately normally distributed (see Multimedia Appendix 2). The interitem correlation analysis showed redundancy between 2 items pertaining to knowledge of resources (ie, “From using Med.Over.Net’s forums, I know where to get information about resources needed to satisfy my health-related needs” and “From using Med.Over.Net’s forums, I know how to get help from others to achieve my health-related goals”; r=0.82, *P*<.001) and 2 items relating to resource mobilization for collective action (“From using Med.Over.Net’s forums, I have realized that the only way to improve health care in our country is by collaborating with other OHC users” and “From using Med.Over.Net’s forums, I feel that I can only impact health care issues by working in an organized way with other OHC users”; r=0.82, *P*<.001). Consequently, we retained only one of the items for each of the above pairs (see Multimedia Appendix 2).

We conducted EFA to obtain communalities for each item and eigenvalues for extracted factors. The EFA showed a solution of 2 factors, whereby 2 items that were both reverse worded had communalities lower than 0.1 and a factor loading of around or below 0.2. Consequently, we omitted both items from further analysis. A principal axis factor analysis was conducted on 11 items of the CE-OHC scale (Multimedia Appendix 2).

The KMO measure was well above the acceptable limit of 0.5 [67] with KMO=0.89, which confirms the sampling adequacy of the analysis. The BTS was also statistically significant (*P*<.001), which indicates that sufficient correlations exist among the variables [68]. The EFA revealed that 2 factors had eigenvalues greater than Kaiser’s criterion of 1 and, in combination, explained around 62.2% of the variance (Multimedia Appendix 2). All items on the CE-OHC scale had communalities around or above 0.4. Cronbach alpha, for both knowledge of resources and resource mobilization for collective action (alpha=.90), indicated good internal consistency. The obtained 2-factor solution was tested using CFA to verify the second-order measurement model of the CE-OHC scale (see Multimedia Appendix 2). The CFA fit indices suggested a reasonably good fit with χ^2^_43_=118.4, CFI=.96, TLI=.95, RMSEA=0.08, and SRMR=0.05. Factor loadings for both subscales were above 0.6, which supports the idea that CE-OHC is a single construct that manifests itself through the knowledge of resources and resource mobilization for collective action (Multimedia Appendix 2). Cronbach alpha (.90) for all items of the CE-OHC scale also supports this solution.

### Testing the Measurement Model: Main Study

Of the 11 items of the CE-OHC scale, item CE-OHC2 had the highest mean (mean 3.72, SD 0.76) and item CE-OHC7 had the lowest mean (mean 2.41, SD 1.12; Table 2). A principal axis factor analysis was conducted on the 11 items using oblique rotation, direct oblimin (Table 2). The KMO measure confirmed the sampling adequacy for the analysis with KMO=0.86, which is well above the acceptable limit of 0.5 [67]. BTS was also statistically significant (*P*=.001), indicating data’s suitability for factor analysis. An initial analysis was run to obtain eigenvalues for each factor in the data, with 2 factors having eigenvalues greater than Kaiser’s criterion of 1 and, in combination, explaining 63.8% of the variance (Table 3). The communalities of all items were greater than 0.3. We retained 2 factors, and the items that cluster on the same factor suggest that the first factor represents *knowledge of resources* and the second factor represents *resource mobilization for collective action*. Cronbach alphas also indicate good internal consistency for both factors (alpha=.87 for knowledge of resources; alpha=.88 for resource mobilization for collective action; Table 3) and the correlation coefficient between factors is r=0.38.

**Table 2:** Factor loadings for collective empowerment in online health communities (CE-OHC) items and descriptive statistics (n=784).

Number of scale items	Scale items (From using Med.Over.Net’s forums…)	Factor 1: Knowledge of resources	Factor 2: Resource mobilization for collective action	Value, mean (SD)
CE-OHC1	*...I know to whom I can turn when I have a health problem.*	0.70	0.02	3.56 (0.90)
CE-OHC2	*...I know how to use the health resources available to me in the OHC.*	0.79	−0.04	3.72 (0.76)
CE-OHC3	*...I know how to get help from others to achieve my health-related goals.*	0.82	−.02	3.55 (0.83)
CE-OHC4	*...I know how to access resources such as information, money, services, or support for dealing with health problems.*	0.74	−0.02	3.20 (0.98)
CE-OHC5	*...I understand better how our country’s healthcare system works.*	0.58	0.07	3.13 (0.97)
CE-OHC6	*...I know which healthcare service I must use to solve my health problems.*	0.72	0.01	3.59 (0.84)
CE-OHC7	*...I actively advocate with other users for better healthcare in our country.*	−0.01	0.65	2.41 (1.12)
CE-OHC8	*...I feel that I can only impact healthcare issues by working in an organized way with other OHC users.*	−0.01	0.79	2.48 (1.11)
CE-OHC9	*...I believe that, to improve healthcare, it is more effective to work with a group of OHC users than as an individual.*	0.01	0.76	3.22 (1.12)
CE-OHC10	*...I realize that only by working together with other OHC users can we muster the power to change the healthcare system.*	0.01	0.87	2.90 (1.08)
CE-OHC11	*...I think that a user becomes powerful in the wider environment only through collaboration with other OHC users.*	0.01	0.79	3.09 (1.06)

**Table 3:** Mean, standard deviation, percentage of variance, and Cronbach alphas of the two factors of collective empowerment in online health communities (CE-OHC) scale.

Factors of CE-OHC scale	Value, mean (SD)	Percentage of variance (%)	Alpha
Factor 1: Knowledge of resources	3.45 (0.68)	42.6	.87
Factor 2: Resource mobilization for collective action	2.82 (0.90)	21.2	.88

**Figure 1:**
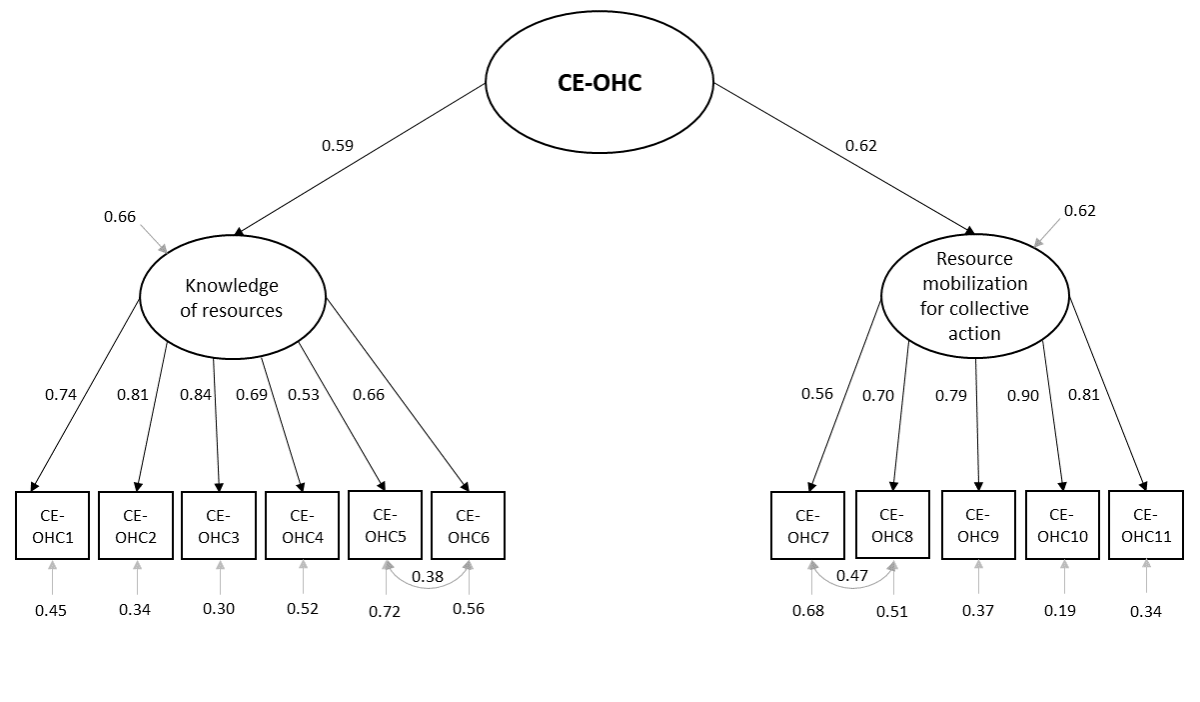
Second-order confirmatory factor analysis of collective empowerment in online health communities (CE-OHC) scale with standardized factor loadings of subscales and their items. CE-OHC1-11: Items of CE-OHC scale.

The 2-factor structure was tested using CFA to inspect the measurement model of the CE-OHC scale. On the basis of the assessment of the modification indices, we freed the covariance between 2 items of *knowledge of resources* (CE-OHC5 and CE-OHC6) and 2 items of *resource mobilization for collective action* (CE-OHC7 and CE-OHC8) as the items use similar phrasing. CFA revealed that the factor loadings for both factors are all above 0.5 (Figure 1). Fit indices with a revised parameter specification gave a better and a reasonably good fit (χ^2^_41_=208.9, CFI=.96, TLI=.95, RMSEA=0.07, and SRMR=0.04), which supports the CE-OHC scale’s 2-dimensional structure (Figure 1). The CE-OHC scale’s good internal consistency was also indicated by Cronbach alpha (.86).

Two subscales were created from the above items, where the knowledge of resources subscale has a higher mean (mean 3.45, SD 0.68) than does resource mobilization for collective action (mean 2.82, SD 0.90; Table 3). For further analysis, we also computed an overall *collective empowerment in OHCs* variable, which was calculated as the average of its subscales.

#### Discriminant and Convergent Validities

To verify the construct validity of the CE-OHC scale, correlation analysis was conducted to test the association among CE-OHC, its subscales, and suggested theoretically (un)related measures, that is, sense of virtual community, intensity of participation, involvement in community organization, and received offline emotional support (Table 4). As hypothesized with regard to convergent validity, the CE-OHC and its subscales, that is, knowledge of resources and resource mobilization for collective action, were significantly correlated with sense of virtual community and intensity of participation, although the correlation between knowledge of resources and intensity of participation is weak (r=0.11; *P*=.003). The results also demonstrated that CE-OHC and its subscale, resource mobilization for collective action, are weakly but significantly associated with involvement in community organization, whereas there is no significant association between the knowledge of resources subscale and involvement in community organization (r=0.02; *P*=.66).

**Table 4:** Bivariate correlations among collective empowerment in online health communities (CE-OHC) scale, its subscales, and theoretically (un)related measures (n=784).

CE-OHC scale and its subscales	Sense of virtual community		Intensity of participation		Involvement in community organization		Received offline emotional support	
	*r*	*P* value	*r*	*P* value	*r*	*P* value	*r*	*P* value
CE-OHC scale	0.44	<.001	0.27	<.001	0.12	.001	0.14	<.001
Knowledge of resources	0.30	<.001	0.11	.003	0.02	.66	0.18	<.001
Resource mobilization for collective action	0.40	<.001	0.30	<.001	0.18	.001	0.07	.06

With regard to the discriminant validity, we expected that the CE-OHC and its subscales would not be associated with received offline emotional support. The results, on the contrary, revealed a statistically significant and weak correlation between CE-OHC and received offline emotional support (*r*=0.14; *P*<.001) and between knowledge of resources and received offline emotional support (*r*=0.18; *P*<.001). As the results presented in Table 4 suggest, only the correlation between received offline emotional support and resource mobilization for collective action was not significant.

### Predictive Validity

To assess the predictive validity of the CE-OHC scale and its subscales, we performed OLS regression. Two regression models were tested: one, in which the CE-OHC scale is included as an independent variable (*single model*), and the other, in which distinct subscales are included as independents variables (*subscale model*). We report the means with standard deviations of all (independent and dependent) variables included in the regression analysis in Table 5, and the results of both regressions are reported in Table 6.

**Table 5:** Descriptive statistics of variables in regression analysis (n=784).

Variables	Value, mean (SD)	Minimum	Maximum
Gender (0=male, 1=female)	0.82 (0.38)	0	1
Age (years)	41.1 (11.5)	18	90
Education	3.67 (0.80)	1	5
Membership length	3.69 (0.60)	1	4
Collective empowerment in online health communities (CE-OHC)	3.14 (0.65)	1	5
Knowledge of resources	3.45 (0.68)	1	5
Resource mobilization for collective action	2.82 (0.90)	1	5
Sense of virtual community	0.72 (0.27)	0	1
Intensity of participation	1.50 (0.62)	1	5
Involvement in community organization	1.15 (0.46)	1	4.33
Civic participation	1.24 (0.51)	1	4.75

**Table 6:** Multiple regression with civic participation as a dependent variable (n=784).

Predictor variables	Single model			Subscales model		
	b^a^	SE	beta	b	SE	beta
Collective empowerment in online health communities (CE-OHC)	0.10	0.02	.13^b^	—^c^	—	—
Knowledge of resources	—	—	—	0.03	0.02	.04
Resource mobilization for collective action	—	—	—	0.07	0.01	.12^b^
Sense of virtual community	−0.08	0.05	−.04^d^	-0.08	0.05	−.04^d^
Intensity of participation	0.13	0.02	.17^b^	0.13	0.02	.16^b^
Involvement in community organization	0.76	0.03	.69^b^	0.75	0.03	.69^b^
Membership length	0.01	0.02	.01	0.003	0.02	.01
Gender	0.02	0.04	.02	0.03	0.04	.02
Age	0.00	0.00	0.007	0.00	0.00	−.01
Education	0.01	0.02	.02	0.01	0.02	.02

^a^b: unstandardized regression coefficient.

^b^*P*<.001.

^c^The empty cells in the table are present because in the single model only collective empowerment in online health communities (CE-OHC) was included as independent variable and in the subscales model subscales knowledge of resources and resource mobilization for collective action were included as independent variables.

^d^.05<*P*<.1.

In the single model, independent variables account for 66.4% of the variance in civic participation, and in the subscale model, independent variables account for 66.5% of the variance of the same dependent variable. The fit of each regression model was significant (F_single_=191.5, *P*<.001; F_subscale_=170.9, *P*<.001). As seen in Table 6, the overall CE-OHC scale demonstrated significant but very weak association with civic participation (beta=.13; *P*<.001). In the subscale model, only resource mobilization for collective action was significantly associated with civic participation (beta=.12; *P*<.001), although the knowledge of resources subscale was not significantly associated with the dependent variable (Table 6).

Among the predictors that were adopted from the literature, intensity of participation in OHCs (beta=.17; *P*<.001) and involvement in community organization (beta=.69; *P*<.001) were significantly associated with civic participation, whereas sense of virtual community was, according to the results (Table 6), very weakly and in the margins of statistical significance related to OHC users’ civic participation. None of the control variables included in the regression models were statistically significantly associated with civic participation (Table 6).

## Discussion

### Principal Findings

The purpose of this study was to conceptualize collective empowerment in OHCs, to develop a scale to measure it, and to inspect the scale’s psychometric properties in terms of factorial structure, reliability, construct validity, and predictive validity.

First, we argued that collective empowerment is, in addition to individual empowerment, an important facet of the concept of patient empowerment with important ramifications for understanding the impact of OHCs on health care in general. Collective empowerment is composed of 2 distinct dimensions (*knowledge of resources* and *resource mobilization for collective action*), which was demonstrated by our pilot and main studies. Our study also showed that the CE-OHC scale may be considered a second-order factor, suggesting that the 11-item scale can be used to measure overall collective empowerment in OHC contexts. The measurement properties of the CE-OHC scale demonstrate that the scale is internally consistent with somewhat limited discriminant and predictive validities.

This study yielded an insight into the construct and predictive validity of the CE-OHC scale. To demonstrate the convergent and discriminant validities, we hypothesized that CE-OHC will be correlated with sense of virtual community, involvement in community organization, and intensity of users’ participation in OHCs but not correlated with received offline emotional support. The results of the main study suggest that evidence for the CE-OHC scale’s convergent validity can be only partially provided. Although the association between the CE-OHC scale (and its subscales) and sense of virtual community, as well as intensity of participation, was confirmed, the correlation between CE-OHC and involvement in community organization was very weak and even absent in the case of the knowledge of resources subscale. In other words, although users may be proactively involved in a community’s activities and organization, it does not correlate with their collective empowerment, in terms of being able to understand the wider sociopolitical contexts of health issues and how to collectively engage to influence such contexts. We may speculate that such a result may be related to the fact that OHCs are complex entities that can include various subcommunities, for example, specific types of online discussion forums such as counseling or support group forums. Such subcommunities usually include specific types of community management, opportunity role structure, and sanctioning and monitoring mechanisms [74]. Involvement in community organization may thus rely heavily on the structure of a specific OHC’s subcommunity, which, crucially, can also affect users’ abilities to use and apply health-related knowledge acquired via the OHC, enabling them to resolve and address health issues within the health care system. In this study, involvement in community organization among OHC users was measured independently from the type of subcommunity or forum (online support group forum or online counseling forum) in which they most often participate. It is recommended that future research should focus on investigating how differences in the specific structural properties of OHC (subcommunities) affect users’ patterns of involvement in online communities and collective empowerment.

Our study did not determine the CE-OHC scale’s discriminant validity to be satisfactory. Offline emotional support is weakly associated with CE-OHC and its subscales, although such associations were not expected. Although research has not hitherto found evidence of a correlation between received offline emotional support and collective empowerment, there may exist a (in)direct link between the fulfillment of caring behaviors and emotional connection among people and their development of collective empowerment. Generally, developed support systems in (online) communities have been identified as an important facilitator of (individual) empowerment [75]. Further studies could focus on identifying the role of exchanged (offline) emotional and other types of social support in arriving at a critical understanding of the sociopolitical environment, knowledge of available resources, and methods of mobilizing those resources to collectively realize goals in the wider public domain. Moreover, future studies could also undertake a multitrait-multimethod approach [76] to assess discriminant and convergent validities and thus focus on investigating the patterns of the relationships between correlations of CE-OHC scale with similar and different constructs using different data collection methods.

In our study, the hypothesis regarding predictive validity was also only partially supported. An overall measure of the CE-OHC scale proved to be a significant predictor of civic participation, although the influence was somewhat weak. The subscale that pertains to knowledge of resources did not have a significant effect on users’ engagement in activities pertaining to issues of public concern. Resource mobilization for the collective action subscale directly pertains to the perception of the collective power developed through an interaction with other individuals (users of the OHCs) and the possibilities of using this power to effect changes in the existing health care system. Thus, the significant direct effect is more plausible than it is in the knowledge of resources subscale, which pertains more to cognitive processes than to action. Although this subscale’s validity is limited, we believe that both subscales present important components of the development of OHC users’ collective empowerment. Resource mobilization is more activation oriented and predominately pertains to an awareness of the power that a collective effort, such as that of an OHC, has. However, resource mobilization also requires knowledge about certain issues, and awareness that these issues cannot be solved individually. Both components are then required for individuals, as members of the community, to gain influence as a whole and, consequently, generate change in the sociopolitical environment’s structure. In the existing literature on collective empowerment, there is a lack of clear evidence of its specific outcomes or its antecedents, which are crucial for defining the nomological network of the collective empowerment construct. This scarcity of research is particularly evident in the health field and in OHC research, which has so far overlooked the importance of these Web-based platforms for the development of collective empowerment and, thus, its investigation. Consequently, the confirmation and validation of our results is currently beyond our reach. The CE-OHC scale will be an important baseline for future research into collective empowerment and should encourage research into this phenomenon and the processes surrounding it in various OHCs.

### Limitations

This study has several limitations, making further research inevitable. In testing the convergent validity of the CE-OHC scale, we were limited by the aforementioned lack of a nomological network that would have offered a stronger theoretical rationale for selecting criteria to establish validity. The evidence for discriminant validity was further hindered for the practical reason that we could not include additional variables in the already lengthy Web questionnaire. The scale’s content validity could also be further improved by the inclusion of a larger set of experts to evaluate the initial pool of items and compute the content validity index.

Further attention should be also given to the optimization of some items’ wording, as 2 pairs of items of each subscale appear to have common content that is disconnected from the content of the latent concept. However, we believe that the reliability of the overall CE-OHC scale, as well as of its subscales, was unaffected by this limitation as the results showed good internal consistency, which was confirmed in both the pilot and main studies.

Another limitation of this study is that both the pilot and main studies were geographically limited and conducted on a single OHC. MON is an OHC that has been in operation for almost 2 decades, and it has a high number and regular base of users and a structure that includes both online support group forums and counseling forums with specifically developed rules and norms that provide a foundation for the development of collective empowerment. Although Slovenia is typical of European Union countries with respect to the internet usage and information communication technologies [77], and MON is comparable with internationally established OHCs, such as PatientsLikeMe, MedHelp, and HealthUnlocked, the development and validation of the scale should include further studies covering different samples, cultures, and OHC settings.

### Implications

This study carries several implications for research and practice. First, we are certain that the CE-OHC scale can be used for a plethora of quantitatively based investigations of emergent phenomena within new information communication technologies. Collective empowerment is an under-researched phenomenon, but it is key to understanding how individual activities transform into a psychological disposition for collective engagement and, in the next step, into actual collective action that has an impact on social change [43]. Explanatory and predictive research requires valid and reliable scales, and it is hoped that the proposal of the CE-OHC scale is a step toward the establishment of widely accepted standardized measures of collective empowerment. Moreover, as OHCs span across different Web-based platforms and include different stakeholders, the scale can be used to measure the collective empowerment of, for example, users of specific Facebook, Discord, Reddit, or Twitter groups, where units of interest may comprise patients, caregivers, and also nurses or doctors.

Second, measuring collective empowerment also carries implications for online community managers. Scales such as the CE-OHC scale allow community managers to assess the emerging social power within OHCs and the nature of this power and to implement measures aimed at managing such activities, for example, by providing functionalities that would help harness such power and providing mechanisms for generating an impact on wider social circumstances. In this context, measuring collective empowerment is also at least indirectly relevant for health policy makers in helping them to identify initiatives or even cases of patient unrest in Web-based platforms. We should note here that we assumed collective empowerment to be *functional*, in that it contributes to positive social change [5]. However, since empowerment can also be dysfunctional [5], this implies that collective empowerment can also be channeled toward unproductive or even damaging goals. Problematic collective behaviors can be evident, for example, in OHCs related to the antivaccination movement [78], proanorexia groups [79], or AIDS-denialist groups [34]. This implies that, when measuring collective empowerment, we should also consider measuring other variables pertaining to the goals of empowerment and other important predictors. Moreover, collective empowerment should be linked to eHealth literacy as previous research [5,59] has shown that a lack of eHealth literacy may lead to dysfunctional empowerment.

### Conclusions

The 11-item CE-OHC scale appears to be a reliable and relatively valid instrument, developed to advance the measurement of collective empowerment in OHC contexts. To the best of our knowledge, the CE-OHC scale represents the first instrument developed for this purpose. The CE-OHC scale can help to identify the potential of OHCs to foster users’ collective engagement and provide a framework that can inform the development of the resources needed to empower OHC social interactions. Research in collective empowerment generally transcends the individualistic approach and contributes to a more holistic understanding of empowerment as a process that brings together individuals, communities, and social change.

## Appendix

Multimedia Appendix 1 Checklist for Reporting Results of Internet E-Surveys.

Multimedia Appendix 2 Pilot study results.

## Abbreviations

BTS: Bartlett test of sphericity
CE-OHC: collective empowerment in online health communities
CFA: confirmatory factor analysis
CFI: comparative fit index
EFA: exploratory factor analysis
eHealth: electronic health
KMO: Kaiser-Meyer-Olkin
MON: Med.Over.Net
OHC: online health community
OLS: ordinary least squares
RMSEA: root mean square error of approximation
SRMR: standardized root mean square residual
TLI: Tucker Lewis Index
