# Supplementary material for: Collective Empowerment in Online Health Communities: Scale Development and Empirical Validation
Source: J Med Internet Res. 2019 Nov 20;21(11):e14392. doi: 10.2196/14392 (PMC6893566; doi:10.2196/14392)
Supplement: Multimedia Appendix 1 [file jmir_v21i11e14392_app1.pdf]

Multimedia appendix 1

Checklist for Reporting Results of Internet E-Surveys (CHERRIES) – CE-OHC scale

Item Category	Checklist item	Explanation
**Design**	Target population	Registered users of Slovenian online health community Med.Over.Net (MON). List-based sampling frame.
	Survey topics	User's experience and satisfaction with MON; Participation and activities in MON; Users' (collective) empowerment outcomes; Users' exchange of social support.
**IRB approval and informed consent process**	IRB approval	As per the code of ethics for researchers at the University of Ljubljana, no institutional ethics approval was needed for this retrospective study. Research was conducted in line with the WMA Declaration of Helsinki on ethical principles for medical research involving human subjects.
	Informed consent	After clicking the link for the Web survey in the email, potential respondents were taken to an informed consent Web page with information about the purpose of research; the length of the survey; an assurance that the data would be dealt with in accordance with national and EU laws; information on who the investigator was and a contact information; and a statement that they were under no obligation to participate and that the aggregated results may be published.
	Data protection	MON is a reputable Web service that treats all personal information (including emails) in accordance with national and EU laws, and protects data with standard security

Item Category	Checklist item	Explanation
		procedures, which include the de-identification of locally held data files, physical protection of hardware, and strong password protection. The OHC provider offered potential respondents the option to participate in a draw for one of five prizes with a total value of 1000€. To enter the draw, respondents needed to disclose personal information, which was processed by the OHC provider, saved separately from the data provided in the survey questionnaire and used strictly for the purposes of the competition. The authors of this study had no access to the emails of respondents and received an anonymized dataset containing no identifiable personal information.
**Development and pre-testing**	Development and testing	The survey was developed in collaboration with the providers of MON as part of their annual survey on user experience and satisfaction with the OHC. Survey development was supported by one survey design specialist at the University of Ljubljana. The online questionnaire testing was conducted by 10 expert evaluators: four experts in social science methodology, two experts in online community and Internet studies, one health communication expert, one online survey methodology expert, one expert in the 1KA online survey tool and one MON user.
**Recruitment process and description of the sample**	Open survey versus closed survey	The survey was open to participants who registered with MON using a valid e-

Item Category	Checklist item	Explanation
**having access to the questionnaire**		mail address and received an email invitation to participate in the Web survey.
	Contact mode	The OHC provider, MON, invited potential respondents to participate in the Web survey via its email newsletter service.
	Advertising the survey	Invitation e-mail advertised the survey as an annual survey on user experiences and satisfaction with the OHC with information on additional module, introduced as a scientific inquiry of the role of OHCs in society. The e-mail invitation was designed in line with recent guidelines for designing e-mail invitations for Web surveys on online community users.
**Survey administration**	Web/E-mail	The survey was administered by the OHC provider through the e-mail newsletter service.
	Context	The e-mail invitation solely contained information on the survey.
	Mandatory/voluntary	Participation in the survey was voluntary.
	Incentives	In exchange for completing the Web survey, potential respondents were by OHC MON provider offered the opportunity to participate in a draw for one of five prizes with a total value of 1000€. The conditions for participation in a prize competition were clearly indicated on the introduction page of the Web survey and on the application form for the prize competition that was included at the end of the Web survey. To enter the draw, respondents had to disclose personal information, which was processed by the OHC provider, saved separately

Item Category	Checklist item	Explanation
		from the data provided in the survey questionnaire and used strictly for the purposes of the competition.
	Time/Date	The survey was administered and available from April 25th to June 10th, 2018.
	Randomization of items or questionnaires	No sets of items were randomized within an items-table. Variables were also not randomized so that the logical structure of the entire questionnaire was retained.
	Adaptive questioning	Where relevant, conditioning and routing were used.
	Number of items	In general, the survey was structured in a format that included one measurement instrument per page. Except in instances where the measurement instrument contained more than 10 items, the table was split in additional pages. The maximum number of items per respondent was 220. The respondents took an average of 21 minutes and 33 seconds to complete the survey.
	Number of screens (pages)	The total number of pages visible to participants was 50 but, due to skip patterns, the total number of pages viewed was lower.
	Completeness check	A completeness check was conducted after the questionnaire was submitted during the analysis phase. The questions did not require a response in order to advance to subsequent questions, except in case of routing questions.
	Review step	Due to the length of the survey, participants were not required to review their responses at survey completion. A “back” button was provided if participants wished to edit previous answers.

Item Category	Checklist item	Explanation
**Response rates**	Unique site visitor	The system in which web survey was administered (http://english.1ka.si/) uses both cookie- and IP-based mechanisms to determine unique site visitor.
	View rate (Ratio of unique survey visitors/unique site visitors)	It was not possible to calculate this.
	Participation rate (Ration of unique visitors who agreed to participate/unique first survey page visitors)	7.71% (2314/30,000)
	Completion rate (Ration of users who finished the survey/users who agreed to participate)	76.15% (1762/2314)
**Preventing multiple entries from the same individual**	Cookies used	Cookies were used and stored for a maximum duration of 1 month. Respondents were informed about cookies in line with the EU cookie policy.
	IP check	IP addresses were not collected from participants, but the survey system collects them and prevents multiple entries from the same IPs.
	Log file analysis	Log file analysis was not performed.
	Registration	Not relevant.
**Analysis**	Handling incomplete questionnaires	Units that provided item nonresponse on the CE-OHC scale used in the analysis were excluded from the study. The remaining units with missing values within the subsample were handled with multiple imputation procedure.
	Questionnaires submitted with an atypical timestamp	Units that had a high level of nonresponse or had dropped out from the survey questionnaire before question Q11 ("How long have you been a user of Med.Over.Net?") were eliminated from the data set. Data from respondents who finished the survey questionnaire in less than

Item Category	Checklist item	Explanation
		five minutes were also eliminated from the data set.
	Statistical correction	No statistical correction procedures or weightings were used in the analysis.
