# Supplementary material for: Collective Empowerment in Online Health Communities: Scale Development and Empirical Validation
Source: J Med Internet Res. 2019 Nov 20;21(11):e14392. doi: 10.2196/14392 (PMC6893566; doi:10.2196/14392)

Multimedia appendix 2

Pilot study results

Table 1. Factor loadings for collective empowerment in online health communities (CE-OHC) items and descriptive statistics (n=280).

Number of scale items	Scale items (From using Med.Over.Net's forums…)	Factor 1: Knowledge of resources	Factor 2: Resource mobilization for collective action	Mean (SD)
CE-OHC1	*…I know to whom I can turn when I have a health problem.*	**0.75**	0.04	3.56 (0.99)
CE-OHC2	*…I know how to use the health resources available to me in the OHC.*	**0.88**	-0.04	3.72 (0.85)
CE-OHC3	*…I know how to get help from others to achieve my health-related goals.*	**0.84**	-0.01	3.49 (0.92)
CE-OHC4	*…I know how to access resources such as information, money, services or support for dealing with health problems.*	**0.77**	-0.03	3.23 (0.98)
CE-OHC5	*…I understand better how our country's healthcare system works.*	**0.56**	0.12	3.15 (1.01)
CE-OHC6	*…I know which healthcare service I must use to solve my health problems.*	**0.77**	0.04	3.56 (0.89)
CE-OHC7	*…I actively advocate with other users for better healthcare in our country.*	0.10	**0.63**	2.90 (0.96)
CE-OHC8	*…I feel that I can only impact healthcare issues by working in an organized way with other OHC users.*	0.03	**0.75**	2.64 (1.00)
CE-OHC9	*…I believe that, to improve healthcare, it is more effective to work with a group of OHC users than as an individual.*	-0.02	**0.82**	3.24 (1.11)
CE-OHC10	*…I realize that only by working together with other OHC users can we muster the power to change the healthcare system.*	-0.07	**0.97**	2.94 (1.05)
CE-OHC11	*…I think that a user becomes powerful in the wider environment only through collaboration with other OHC users.*	0.11	**0.74**	3.07 (1.07)

Table 2. Mean, standard deviation, percentage of variance and Cronbach alphas of the two factors of collective empowerment in online health communities (CE-OHC) scale.

Factors of CE-OHC scale	Mean (SD)	Percentage of variance	Alpha
Factor 1: Knowledge of resources	3.46 (0.75)	33.1	.90
Factor 2: Resource mobilization for collective action	2.97 (0.87)	29.1	.90

Figure 1. Second-order confirmatory factor analysis of collective empowerment in online health communities (CE-OHC) with standardized factor loadings of subscales and their items on data collected in the pilot study. CE-OHC1-11: Items of CE-OHC scale.

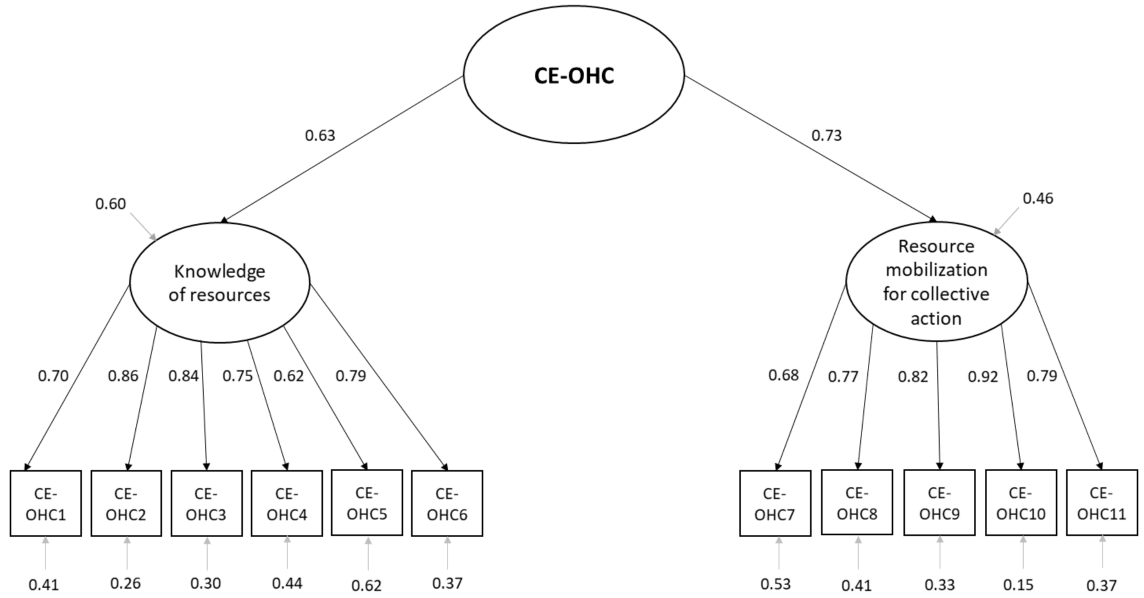

Supplement: Multimedia Appendix 2 [file jmir_v21i11e14392_app2.pdf]
